# Short developmental milestone risk assessment tool to identify Duchenne muscular dystrophy in primary care

**DOI:** 10.1186/s13023-024-03208-8

**Published:** 2024-05-10

**Authors:** Paula van Dommelen, Oisín van Dijk, Jeroen A. de Wilde, Paul H. Verkerk

**Affiliations:** 1 Department of Child Health, The Netherlands Organization for Applied Scientific Research TNO, Leiden, The Netherlands; 2 Department of Public Health and Primary Care, Leiden University Medical Center, Leiden, The Netherlands

**Keywords:** Pediatrics; neuromuscular disorder, Child Development, Developmental Delay, Early Childhood Development, Motor skills

## Abstract

**Background:**

In patients without a family history, Duchenne muscular dystrophy (DMD) is typically diagnosed at around 4–5 years of age. It is important to diagnose DMD during infancy or toddler stage in order to have timely access to treatment, opportunities for reproductive options, prevention of potential fatal reactions to inhaled anesthetics, awareness of a child’s abilities needed for good parenting, and opportunities for enrolment in clinical trials.

**Method:**

We aimed to develop a short risk assessment tool based on developmental milestones that may contribute to the early detection of boys with DMD in primary care. As part of the case-control 4D-DMD study (Detection by Developmental Delay in Dutch boys with DMD), data on developmental milestones, symptoms and therapies for 76 boys with DMD and 12,414 boys from a control group were extracted from the health records of youth health care services and questionnaires. Multiple imputation, diagnostic validity and pooled backward logistic regression analyses with DMD (yes/no) as the dependent variable and attainment of 26 milestones until 36 months of age (yes/no) as the independent variable were performed. Descriptive statistics on symptoms and therapies were provided.

**Results:**

A tool with seven milestones assessed at specific ages between 12 and 36 months resulted in a sensitivity of 79% (95CI:67–88%), a specificity of 95.8% (95%CI:95.3–96.2), and a positive predictive value of 1:268 boys. Boys with DMD often had symptoms (e.g. 43% had calf muscle pseudohypertrophy) and were referred to therapy (e.g. 59% for physical therapy) before diagnosis.

**Discussion:**

This tool followed by the examination of other DMD-related symptoms could be used by youth health care professionals during day-to-day health assessments in the general population to flag children who require further action.

**Conclusions:**

The majority of boys (79%) with DMD can be identified between 12 and 36 months of age with this tool. It increases the initial a priori risk of DMD from 1 in 5,000 to approximately 1 in 268 boys. We expect that other neuromuscular disorders and disabilities can also be found with this tool.

**Supplementary Information:**

The online version contains supplementary material available at 10.1186/s13023-024-03208-8.

## Background

Worldwide, health care professionals often use monitoring tools to test the developmental skills of infants and toddlers [1, 2]. An important goal of monitoring child development is the early identification of a wide range of disorders that impact child development. Typically, ‘red flags’ for milestone attainment are set at approximately the 90th percentile, i.e. with 90% of children attaining the milestone. However, if a child fails to attain a milestone, it is still uncertain if and to what extent the risk of a disorder is increased. For many disorders it is unknown how the monitoring tools can be optimally used to have a high sensitivity and specificity at field level.

One of such disorders is Duchenne muscular dystrophy (DMD). DMD is an inherited X-linked recessive neuromuscular disorder affecting approximately 1 in 5000 live male births [3, 4]. DMD is typically diagnosed at around 4–5 years of age [5–7]. It is important to diagnose DMD during infancy or at the toddler stage in order to have timely access to treatment [8, 9], opportunities for reproductive options, prevention of potential fatal reactions to inhaled anesthetics [10], awareness of a child’s abilities needed for good parenting, and opportunities for enrolment in clinical trials [11]. 

Previous studies have shown that more children with DMD fail to attain some developmental milestones compared to the general population [12–17]. Studies also recognized diagnostic delay despite parents noticing signs and symptoms in their child that are characteristic of DMD [5]. Several risk assessment tools were reported including developmental milestones for DMD [5, 18, 19]. These tools suggest performing a serum creatine kinase (CK) test if a child is unable to walk at 16–18 months [5, 18, 19], shows Gowers’ sign [19], or does not use at least ten recognizable words at 24 months of age [18]. However, the diagnostic validity of these tools was not assessed. Therefore, the tools do not indicate the increased risk of DMD given a developmental delay.

Our previous research investigated the diagnostic validity of a large number of individual milestones and showed that the milestones ‘walks well alone at 24 months’ and ‘walks smoothly at 36 months’ were most promising in detecting boys with DMD [17]. However, a tool that uses combinations of milestones may improve the diagnostic validity. Since there is a wide variation in the selection of milestones and the timing of their use worldwide, a short tool is needed to implement this in the primary care workflow to improve the early detection of DMD.

The aim of this study is to develop a short risk assessment tool based on developmental milestones for the early detection of DMD with acceptable diagnostic properties that can be easily applied during day-to-day health assessments in the general population.

## Methods

### Data collection

Within the 4D-DMD study (Detection by Developmental Delay in Dutch boys with DMD) with a case-control design, data were collected from: (1) health records of boys with DMD; characteristics, referrals to secondary and tertiary care, educational interventions, clinical descriptions typical of DMD, and developmental scores; (2) questionnaires completed by parents of boys with DMD; type of diagnosis, recall of developmental milestones, health care referrals, symptoms, concerns; and (3) health records of a control group of a general population of boys from the Youth Health Care (YHC) of The Hague (one boy with diagnosed DMD was excluded); characteristics, developmental scores, and referrals to other health professionals.

The diagnosis and date of diagnosis were obtained from the Dutch DMD patient registry. More information about the data collection within the 4D-DMD study is available in our previous research [17]. 

### Developmental milestones

In the Netherlands, there is a well-organized YHC system, where 95% of all children are seen at regular visits [20]. Basic care within the Dutch YHC is supported by 35 evidence-based guidelines and validated screening tools [21], facilitating referrals as necessary. In the Netherlands, the Dutch Development Instrument (DDI) [22], a modification of the Gesell test, is used by YHC to assess the development of children. The DDI is mentioned in seven YHC guidelines, and among these, one guideline is dedicated to language development and one to motor development. However, none of these guidelines specifically address DMD. The DDI is a set of 75 developmental milestones that cover three domains of child development: (1) fine motor activity, adaptive behaviour, and personal/social behaviour; (2) communication; and (3) gross motor activity. The DDI is administered by trained YHC professionals at visits scheduled at the ages of 1, 2, 3, 6, 9, 12, 15, 18, 24, 30, 36, 42, and 48 months. For this study, we selected milestones up until 36 months of age. In many Dutch YHC services visits at 30 months are only scheduled for children considered at risk. Therefore, milestones registered during this visit were excluded. YHC professionals administer and register each milestone according to a uniform protocol. Two to seven specific milestones are registered in the health records at each visit. Some milestones may also be registered based on observations made by caregivers if the behaviour is not observed during the examination.

### Statistical analysis

To develop the short risk assessment tool to identify boys with DMD that could easily be used in daily practice of primary care, we needed to determine which and to what extent the developmental milestones independently contribute to the risk of DMD. We applied the following six steps:

1. *Pre-selection of data*
  Previous research within the 4D-DMD study showed that 26 milestones between 2 and 36 months were univariate significant at 0.01 level or lower between the DMD and control group [17]. For this study, we selected these 26 milestones to reduce the number of variables for the imputation in step 2, because the sample size in the DMD group does not allow a large number of variables.
2. *From incomplete to complete data*
  Multiple imputation was applied in both groups (DMD, control) to predict missing data in the 26 milestones (see appendix for the observed and missing values) [23]. In total, 50 predictions were conducted to account for missing data uncertainty.
3. *Models to obtain selection of milestones for the short risk assessment tool*
  We developed five age-dependent models for the early identification of DMD using milestones up until (1) 12 months, (2) 15 months, (3) 18 months, (4) 24 months, and (5) 36 months of age. For each prediction, logistic regression analyses were performed and afterwards pooled to test the impact of the milestones (independent variables) on group (DMD vs. control) outcome. Backward stepwise regression was applied on the pooled models till all remaining variables were significant at 0.05 level in the final model. We selected milestones that were statistically significantly associated with the outcome (DMD yes/no) in one or more of the final age-dependent models. Milestones that were not significant in all models (but significant in at least one model) were also taken into account, because these milestones may reduce the age of detection.
4. *From model parameters to simple weighing factors*
  In order to create one practical tool that can be easily implemented in daily practice, we investigated whether simple weighting factors with integer numbers can be used instead of employing computer-intensive regression models. We tried several weighting factors (1 to 13) for each selected milestone from step 3 and calculated the sum score after weighting each milestone (with 1 point for a fail on a milestone and 0 points for a pass on a milestone or a when a milestone is not assessed) to achieve the highest predictive value. Note that a higher weight for a milestone implies a greater likelihood that the boy has DMD when the boy fails this milestone.
5. *Predictive value of the cut-off values for the sum score*
  We then applied cut-off values for the sum score to calculate the sensitivity (% of referrals according to the tool within the DMD group) and specificity (% of non-referrals according to the tool within the control group), and the positive predictive value (PPV: how many boys with DMD are available within the referrals according to the tool assuming a prevalence of 1:5000 live male births). The negative predictive value (NPV: how many controls are available within the non-referrals according to the tool assuming a prevalence of 1:5000 live male births) was not calculated, because the prevalence of DMD is low and results in a NPV of almost 100%.
6. *Selection of optimal cut-off values for the sum score*
  We obtained the most optimal weighting factors and cut-off value by choosing the highest sensitivity at a fixed specificity of approximately 95%. As a condition, the weighting factor for the milestone walks smoothly at 36 months was set at the highest cut-off value, because of the high risk of DMD. Also, up until 15 months of age, failures of at least two milestones were selected to reduce the number of false-positives at an early age.

All analyses were conducted in R Version 3.4.4 and SPSS Version 25.

## Results

The parents of 229 boys with DMD who met the inclusion criteria were invited to participate. In total, 87 boys with DMD and/or their parents gave written permission for retrieval of their health records. Retrieval was unsuccessful in ten cases: data were missing or not available for nine and one boy did not survive during retrieval of his records. In total, the health records of 76 boys with DMD were received. In addition, 71 parents of boys with DMD fully or partly completed the questionnaire.

Epidemiological and disease characteristics of boys with DMD and the general population are summarized in Table 1. The proportions of boys with DMD (cases) and boys without DMD (controls) who failed the developmental milestones at each age in the observed (YHC and Questionnaire) and imputed data (YHC) are shown in the appendix.

**Table 1:** General characteristics of boys with Duchenne muscular dystrophy (DMD) and boys in the control group

Characteristics	DMD YHC^a^ (*N* = 76)		DMD YHC^a^ +Q^b^ (*N* = 104)		Control group (*N* = 12,414)	
	*N*	Mean (SD) or %	*N*	Mean (SD) or %	*N*	Mean (SD) or %)
Gestational age (weeks)	66	39.1 (2.3)	96	39.2 (2.3)	11,509	38.9 (1.9)
Birth weight (grams)	69	3400 (760)			11,550	3,399 (582)
Age at diagnosis (in years)						
*Total*	76	4.0 (2.0)	103	4.1 (2.0)		
*No family history of DMD*	*68*	*4.3 (1.9)*				
*Known family history of DMD*	*6*	*1.5 (1.2)*				
*Family history of other neuromuscular disease*	*2*	*1.4 (1.3)*				
Type of diagnosis						
*Deletion in DMD-gene*	25	*63*	*40*	*63*		
*Insertion in DMD-gene*	8	*20*	*12*	*19*		
*Small or other mutation*	7	*18*	*12*	*19*		

^a^Data obtained from the Youth Health Care (YHC) files, ^b^Data obtained from the questionnaire filled out by parents from cases with DMD (Q). Columns 1–3, 6–7 are adapted from ‘van Dommelen P, van Dijk O, Wilde JA, Verkerk PH. Early developmental milestones in Duchenne muscular dystrophy. Dev Med Child Neurol 2020;62: 1198–1204’

A total of 570 referrals to 45 different healthcare providers or pedagogical interventions were extracted from the YHC records with a mean result of 7.5 referrals per boy with DMD. We combined data when data were available from both the YHC records and the Questionnaire (Q). A high number of undiagnosed boys with DMD were already referred to physiotherapy (26% aged 0-0.99y and 39% aged 1-3.99y, speech-language therapist (17%), Ear-Nose-Throat (ENT)-specialist (16%, YHC data) and preschool educational intervention (9%, YHC data). Symptoms that appeared often in DMD boys were pseudohypertrophy of the calf muscles (43%), falling more frequently compared to peers (27%, YHC data), stiff gait (19%, YHC data), a younger appearance than his chronological age (which may be related to behaviour and/or growth) (11%, YHC data). Between 0-3.99y, three in four parents of boys with DMD (77%, Q data) had concerns about their child’s developmental delay, mainly concerning their motor skills (85% out of concerned parents). Between 0-3.99y, approximately one in ten undiagnosed boys with DMD (11%, Q data) required surgery and were exposed to inhalational agents during surgery.

Table 2 shows the results from the five age-dependent pooled logistic regression models after stepwise backward regression on the developmental milestones. The footnote of Table 3 provides a detailed description of each milestone. Independent predictors of DMD were failing for ‘pulls up to standing position’, ‘reacts to a verbal request’, and ‘sits in stable position without support’ at 12 months, ‘crawls abdomen off the floor’ at 15 months, ‘walks alone’ at 18 months, ‘walks well’ at 24 months, and ‘walks smoothly’ at 36 months. Milestones before the age of 12 months were not statistically significant after adjustment for the milestones at 12 months of age. In total, seven milestones were independent predictors of DMD. As these models (with different weighing factors and an exponential component) are not easy to use in daily practice, we simplified the weighing factors (with integer numbers and a linear instead of an exponential component) in the next step of the analysis using these seven milestones.

**Table 2:** Results from the pooled logistic regression models after stepwise backward regression on the developmental milestones

	12 months B (SE)^a^	12–15 months B (SE)^a^	12–18 months B (SE)^a^	12–24 months B (SE)^a^	12–36 months B (SE)^a^
**Model**					
Intercept^b^	-6.12 (0.22)	-6.20 (0.22)	-6.21 (0.22)	-6.15 (0.22)	-6.30 (0.23)
Fails to pull up to standing position at 12 months	2.24 (0.37)***	1.59 (0.46)***	1.44 (0.50)**	1.71 (0.45)***	1.39 (0.50)**
Fails to react to a verbal request at 12 months	1.27 (0.45)**	1.19 (0.48)*	1.30 (0.44)**		1.42 (0.69)*
Sits in stable position without support at 12 months	1.31 (0.36)***	0.79 (0.39)*			
Fails to crawl abdomen off the floor at 15 months		1.94 (0.45)***	1.68 (0.49)***	1.58 (0.48)**	1.97 (0.53)***
Fails to walk alone at 18 months			1.09 (0.47)*		
Fails to walk well alone at 24 months				2.50 (0.39)***	
Fails to walk smoothly at 36 months					4.72 (0.90)***

^a^The estimated effects (B) instead of the odds ratios are provided, such that the predicted probability of Duchenne muscular dystrophy can easily be calculated with the model: EXP(sum score)/(1 + EXP(sum score)). ^b^For the intercepts a correction of log(1/30.6106)=-3.42 in needed as the ratio of the number of boys with Duchenne muscular dystrophy versus controls in our sample was a factor 30.6106 higher compared to the ratio in the general population assuming a prevalence of 1:5000 live male births. This is the quantity you add to the intercept of the models. Note that the B’s are the weighing factors for each milestone (and the intercept); a higher B implies a higher risk of Duchenne muscular dystrophy

^*^p < 0.05. **p < 0.01. ***p < 0.001

**Table 3:** Diagnostic properties at various cut-off point for the sum scores of the short tool to detect Duchenne muscular dystrophy (DMD)

Short tool (Failure = 1, Pass or not assessed = 0): Sum score = 1 x Pulls up to standing position at 12 months^a^ + 2 x Reacts to a verbal request at 12 months^b^ + 2 x Sits in stable position without support at 12 months^c^ + 2 x Crawls abdomen off the floor at 15 months^d^ + 1 x Walks alone at 18 months^e^ + 4 x Walks well alone at 24 months^f^ + 13 x Walks smoothly at 36 months^g^ For example, a boy aged 15 months fails to pull up to a standing position (score of 1), passes for reacts to a verbal request (score of 0), fails to sit in a stable position without support (score of 1), and fails to crawl with the abdomen off the floor (score of 1). The scores for milestones > 15 months are 0. The sum score is then 1 × 1 + 2 × 0 + 2 × 1 + 2 × 1 + 1 × 0 + 4 × 0 + 13 × 0 = 5, which is ≥ 3. In this case, further clinical investigation of the boy is recommended.					
Cut-offs for the sum score	Sensitivity (%, 95% CI)	Specificity (%, 95% CI)	Positive predictive value	Sensitivity at 12 months (%)	Sensitivity between 12–24 months (%)
≥ 3	79 (67–88)	95.8 (95.3–96.2)	1:268	40 (28–54)	71 (58–81)
≥ 4	69 (57–80)	97.4 (97.0-97.7)	1:191	14 (6–27)	60 (47–72)
≥ 5	64 (52–75)	98.6 (98.3–98.8)	1:112	11 (5–24)	53 (41–65)
≥ 6	58 (46–69)	98.9 (98.6–99.1)	1:98	0	42 (31–55)
≥ 7	48 (36–61)	99.4 (99.2–99.5)	1:66	0	29 (19–42)
≥ 9	42 (30–55)	99.6 (99.5–99.8)	1:51	0	14 (7–26)
≥ 11	37 (25–50)	99.8 (99.6–99.9)	1:31	0	5 (2–15)
≥ 13	34 (22–47)	99.9 (99.7–99.9)	1:19	0	0

^a^The boy finds it difficult pulling up to a standing position (sometimes one hand on ‘something’ and one hand on the knee) or cannot raise himself up to stand (or the boy is constantly standing on tiptoe) and the parent has also not observed standing up at the age of 12 months. ^b^The boy does not respond by pointing or looking in the direction of an object to which the person is asked and also the parent did not observe this at the age of 12 months. ^c^The boy cannot get loose or becomes unbalanced when following an object or almost falls if it is brought out of balance, because he makes no or insufficient recovery movements and/or does not use his hands as support at the age of 12 months. ^d^The boy does not crawl forwards with the abdomen off the floor, but crawls with the abdomen on the floor (forwards or backwards) or moves bottom-shuffling or rolls or does not move at all and the parent has not observed crawling at the age of 15 months. ^e^The boy does not walk at least three steps (or more than half of the time on his toes), has a lordotic posture, stiffy mannered way of walking, falls more frequently compared to peers and/or abnormal or clumsy gait at the age of 18 months. ^f^The boy does not walk or less than 5 m (or more than half the time on his toes), repeatedly loses his balance when walking and is unable to absorb his fall, has a lordotic posture, wide gait, stiffy mannered way of walking, falls more frequently compared to peers and/or abnormal or clumsy gait at the age of 24 months. ^g^The boy has a lordotic posture, wide gait, asymmetrical and stiffy mannered way of movements of legs, torso and shoulders, arms are not smoothly moved from the shoulders in the opposite direction to the contralateral leg, the boy cannot avoid objects in the room when walking, he walks mostly on his toes, repeatedly loses his balance and cannot prevent a fall and/or has an abnormal or clumsy gait at the age of 36 months

Table 3 shows the results of the most optimal weighting factors and diagnostic value for the independent predictors of DMD. A higher sum score increased PPV and specificity, but decreased sensitivity. With this tool and a cut-off of 3 for the sum score, approximately eight out of ten boys may be identified by their development between 12 and 36 months of age and seven out of ten boys between 12 and 24 months of age. Further analyses on patients by mutation type revealed that the detection rate of the tool with a cut-off of 3 for the sum score was 73% in patients with a deletion in DMD-gene (*n* = 40), 73% with an insertion in DMD-gene (*n* = 12), 64% with a small or other mutation (*n* = 12) and 88% in patients for whom the type of mutation was unknown.

## Discussion

The main finding of our study was that a combination of developmental milestones (six gross motor activity and one communication) assessed at specific ages may be a useful tool for primary care to identify boys at increased risk of DMD. Our study shows that the tool has the potential to detect eight in ten boys with DMD between 12 and 36 month of age. A sum score of ≥ 3 according to the tool increases the initial a priori risk of DMD from 1 in 5,000 to approximately 1 in 268 boys. Other findings of our study are that undiagnosed boys often had symptoms (e.g. 43% had calf muscle pseudohypertrophy) and were referred to therapy (e.g. 59% for physical therapy).

Important factors when choosing values for sensitivity and specificity of the tool include the prevalence and severity of the disease, the consequences of not detecting the disease, the importance of early detection and avoiding needless parental concern. In the recommendations on developmental screening tests from the American Academy of Pediatrics, sensitivity and specificity levels of 70–80% are considered acceptable [24]. In our study we selected higher specificity levels, because a low prevalence in combination with a relatively low specificity results in a low PPV. Therefore, we decided to develop a risk assessment tool instead of a screening tool, because the majority of disorders with a low prevalence cannot easily be found with factors others than blood or gene tests. However, in the case of developmental delay, other disorders that impact development may also be included in the prevalence. In total, 0.16% of all children have a neuromuscular disorder [25] and 5% have some type of moderate to severe disability [26]. We have, therefore, selected a minimum specificity of 95%. For many of these children, further investigation of the developmental delay may be helpful, because our previous research showed that disorders that impact development cannot always be regarded as isolated disorders [17, 27]. 

With the present system, many boys with DMD are detected later than desired. Implementation of this tool in the Netherlands may improve this. Our tool is constructed in such a way that it can be easily implemented in other health care systems. Several of the milestones in the short risk assessment tool (not able to walk at 18 months [5, 18, 19]) and further specifications (weakness, toe walking, abnormal or clumsy gait, frequent falls [12, 18, 19]) were also mentioned in the literature. More risk factors were previously found in other studies such as Gowers’ sign, difficulty climbing stairs [5, 12, 19], painful legs or joints [18], and the presence of non-motor delay such as delayed speech and language acquisition [12, 13, 18, 19], poor cognition or behaviour problems [28]. Moreover, growth failure and obesity were reported more often in boys with DMD [29].

Taken all this information into account, we have several recommendations for the early detection of DMD.

### Recommendations for practical use of the tool

The tool with the seven milestones (see Table 3) could be used by YHC professionals during day-to-day health assessments in the general population to flag children who require further action. Further investigation into the presence of symptoms for neuromuscular disorders or disabilities is needed.

Our study found that several symptoms were often reported. The following questions may, therefore, be relevant to investigate if the child (in this case a boy) has a sum score ≥ 3 according to the tool:

- A family history of neuromuscular disease?
- Any presence of DMD-specific symptoms (calf muscle pseudohypertrophy, stiffy gait, falls more frequently compared to peers, appears to be younger than his chronological age)?
- Attend therapy for his motor and/or speech delay (physical, speech-language)? Visited an ENT-specialist?
- Parental concerns about their child’s motor (and speech) delay?
- Failures on other milestones (shown in the Appendix)?

Literature shows that other questions may also be relevant [5, 12, 18, 19, 28–32].

- Increased head circumference, failure to thrive, overweight?
- Difficulty with stair climbing?
- Difficulty with running?
- Inability to jump?
- Decreased endurance?
- Weakness of the proximal muscles (has to use their hands and arms to “walk” up their own body from a squatting position: Gowers’ sign)?
- Toe walking?
- Flat feet?
- Inability to keep up with peers?
- Painful legs or joints?
- Cognitive delay?
- Learning and attentional issues?
- Behaviour issues?
- Autism spectrum disorder?

We recommend YHC professionals to register information from these questions, as well as data from other health care providers involved with the child, in the electronic health records. When the investigation is complete, one may decide to wait and monitor the development closely or consider CK testing, because CK is extremely elevated (50- to 200-fold above normal levels [5]) in boys with DMD and it is a relatively cheap and fast test. Especially in the situation where there are concerns, either by the parents or by one or more health care providers, we recommend a CK test. High levels of CK prompts referral to a pediatric neurologist, with input from a geneticist or genetic counsellor, to prevent diagnostic delay [5]. However, even with normal levels of CK, referral to a pediatric neurologist or other specialists may be necessary to reduce diagnostic delay in other neuromuscular disorders or some other type of developmental disability such as cerebral palsy, non-syndromic intellectual developmental disorder and autism. In view of the current incurability, the progressive course and the always fatal outcome of DMD, the most important therapeutic task in the early course of DMD is the medical, psychosocial and genetic counselling of families.

The tool should not be promoted as a screening tool for DMD, due to its relatively low positive predictive value, the potential for yielding abnormal results for other conditions besides DMD, and to avoid stress among families. It is important to investigate the adoption and acceptability of the tool before proceeding with implementation. One of the aspects that requires attention is the naming of the tool without emphasizing the condition DMD.

Compared to newborn screening (NBS) where CK levels are evaluated in the first screen, an advantage of this approach would be that a smaller group undergoes testing, and avoids the potential problem of NBS of elevated CK levels being elevated in newborns due to birth trauma [33]. A disadvantage is that approximately two in ten boys with DMD cannot be identified by the tool, and the tool will lead to false-positive results, although some of these may have another disorder that impact development. Moreover, our study shows that approximately one in ten undiagnosed boys with DMD had an increased risk of detrimental consequences due to the exposure to inhalational agents during surgery before they were four years of age. To prevent such risks, and given advances in diagnostics and promising therapeutic approaches, the discussion on inclusion of DMD in NBS should be continued.

### Strengths and limitations

A strength of our study is that milestones were determined during real-world regular day-to-day health assessments in the general population. This increases the generalizability of our tool for use in daily practice. Furthermore, YHC professionals were mainly blinded for the diagnosis because most of the data were registered before the diagnosis of DMD was made. A limitation is that the number of observations varied between milestones and visits. Although YHC in the Netherlands is highly standardized, parents do not always attend all visits when their child is between 1 and 36 months of age. Also, health care professionals do not always register all milestones during a visit, partly, we believe, attributable to time pressure in YHC practice. However, approximately the same attendance rates and the same registration method occurred for both the DMD and the control groups. Moreover, we applied multiple imputation to adjust for missing values. Another limitation is that we were unable to explore the likelihood of referral within the current YHC setting due to the potential for concerns to arise from various sources, including YHC, parents/caregivers, childcare facilities, general practitioners, or others.

## Conclusions

Our short risk assessment tool, which was based on combinations of developmental milestones at specific ages, combined with symptoms and referrals to therapy could be helpful in identifying boys with DMD. This tool is quick and easy to implement. A major advantage would be that it could enable the majority of boys (79%) with DMD to be identified between 12 and 36 months of age, and 71% between 12 and 24 months. We expect that other neuromuscular disorders and disabilities can also be found with this tool. With preparation and investigation into its adoption and acceptability, this tool can be integrated into the workflow of primary care practices [34]. Using a validated risk assessment tool at regular, repeated intervals, in addition to physician surveillance at well-child visits, may improve early detection [30]. We recommend more research with new datasets to validate the tool.

### Electronic Supplementary Material

Below is the link to the electronic supplementary material.

Supplementary Material 1

## Data Availability

It is not possible to share research data publicly, because individual privacy could be compromised.
