# Supplementary material for: Short developmental milestone risk assessment tool to identify Duchenne muscular dystrophy in primary care
Source: Orphanet J Rare Dis. 2024 May 10;19:192. doi: 10.1186/s13023-024-03208-8 (PMC11088161; doi:10.1186/s13023-024-03208-8)
Supplement: Supplementary file 1 — Supplementary Material 1 [file 13023_2024_3208_MOESM1_ESM.docx]

Appendix. Proportion of boys with Duchenne muscular dystrophy (DMD) and boys in the control group who failed to attain developmental milestones at specific ages

|  |  | DMD  Observed data YHC | | | DMD  Imputed data YHC  (n=76) | DMD Question-  naire^c^ | |  | Control group  Observed data | | | Control group Imputed data (n=12,414) |  | DMD vs control group  Imputed data |
| --- | --- | --- | --- | --- | --- | --- | --- | --- | --- | --- | --- | --- | --- | --- |
| Age^a^  (mo) | Milestones (Domain^b^) | Age at visit (mo)  Mean (SD) | Fail  n/N | Fail  % | Fail  % | Fail  n/N | Fail  % |  | Age (months)  mean (SD) | Fail  n/N | Fail  % | Fail  % |  | OR^d^ (95%CI) |
| 2 | Smiles in response (C) | 2.2 (0.6) | 4/63 | 6 | 7 |  |  |  | 2.2 (0.5) | 146/8,859 | 1.6 | 1.9 |  | 4.1 (1.5-11.1)** |
| 2 | Follows with eyes and head 30° < 0° > 30° (F) | 2.3 (0.6) | 13/63 | 21 | 21 |  |  |  | 2.2 (0.5) | 860/8,533 | 10.1 | 10.3 |  | 2.3 (1.3-4.3)** |
| 3 | Lifts head to 45° in prone position (G) | 3.1 (0.4) | 14/63 | 22 | 25 |  |  |  | 3.2 (0.5) | 944/9,069 | 10.4 | 10.6 |  | 2.8 (1.5-5.1)** |
| 9 | Rolls over back and forth (G) | 9.1 (0.7) | 5/57 | 9 | 12 |  |  |  | 8.7 (0.8) | 250/8,893 | 2.8 | 2.9 |  | 4.4 (1.8-10.4)** |
| 9 | Plays with both feet (F) | 9.2 (0.7) | 3/54 | 6 | 6 |  |  |  | 8.8 (0.9) | 84/7,384 | 1.1 | 1.2 |  | 5.3 (1.8-16.3)** |
| 9 | Balances head well while sitting (G) | 9.1 (0.6) | 4/58 | 7 | 6 |  |  |  | 8.7 (0.8) | 99/8,891 | 1.1 | 1.2 |  | 5.5 (1.9-15.7)** |
| 9 | Sits on buttocks while legs stretched (G) | 9.2 (0.7) | 8/54 | 15 | 18 |  |  |  | 8.8 (0.8) | 488/8,454 | 5.8 | 5.9 |  | 3.6 (1.8-7.1)*** |
| 12 | Crawls forward abdomen on the floor (G) | 11.4 (0.6) | 22/55 | 40 | 41 | 34/66 | 52 |  | 11.3 (0.6) | 487/7,112 | 6.8 | 6.2 |  | 10.7 (6.4-17.9)*** |
| 12 | Pulls up to standing position (G) | 11.4 (0.6) | 35/51 | 69 | 64 | 40/64 | 63 |  | 11.3 (0.5) | 753/8,645 | 8.7 | 9.3 |  | 17.1 (9.8-31.8)*** |
| 12 | Waves "bye-bye" (C) | 11.5 (0.7) | 17/50 | 34 | 38 |  |  |  | 11.2 (0.4) | 924/8,154 | 11.3 | 11.5 |  | 4.7 (2.7-8.1)*** |
| 12 | Picks up pellet between thumb and index finger (F) | 11.4 (0.6) | 9/49 | 18 | 22 |  |  |  | 11.2 (0.4) | 613/7,772 | 7.9 | 8.0 |  | 3.1 (1.7-6.0)*** |
| 12 | Sits in stable position without support (G) | 11.4 (0.7) | 21/57 | 37 | 38 | 21/63 | 33 |  | 11.3 (0.6) | 248/8,193 | 3.0 | 3.0 |  | 19.9 (11.5-34.2)*** |
| 12 | Reacts to a verbal request (C) | 11.5 (0.5) | 7/41 | 17 | 24 |  |  |  | 11.2 (0.4) | 176/7,990 | 2.2 | 2.3 |  | 13.2 (6.5-26.9)*** |
| 15 | Crawls abdomen off the floor (G) | 14.6 (0.6) | 28/55 | 51 | 49 | 32/62 | 52 |  | 14.2 (0.5) | 237/7,840 | 3.0 | 2.9 |  | 32.6 (18.8-56.3)*** |
| 15 | Walks along/cruising (G) | 14.5 (0.5) | 23/57 | 40 | 42 | 28/68 | 41 |  | 14.2 (0.5) | 290/7,638 | 3.8 | 3.5 |  | 20.2 (12.1-33.6)*** |
| 18 | Says three “words” (C) | 18.9 (1.5) | 13/43 | 30 | 36 |  |  |  | 18.3 (0.8) | 975/8,107 | 12.0 | 12.1 |  | 4.0 (2.2-7.2)*** |
| 18 | Walks alone (G) | 18.5 (1.6) | 28/52 | 54 | 52 | 29/67 | 43 |  | 18.3 (0.9) | 378/8,137 | 4.6 | 4.8 |  | 21.5 (12.6-36.4)*** |
| 24 | Walks well alone (G) | 24.5 (1.5) | 22/64 | 34 | 36 | 38/59 | 64 |  | 24.8 (1.3) | 54/8,112 | 0.7 | 0.8 |  | 72.5 (41.3-127.1)*** |
| 24 | Imitates others (F) | 24.9 (1.2) | 3/52 | 6 | 5 |  |  |  | 24.9 (1.1) | 74/7,838 | 0.9 | 1.1 |  | 4.6 (1.4-15.2)* |
| 24 | Says ‘sentences’ of two words (C) | 24.7 (1.4) | 23/57 | 40 | 41 | 5/56 | 9 |  | 24.9 (1.2) | 1,664/8,357 | 19.9 | 20.0 |  | 2.9 (1.6-4.6)*** |
| 24 | Squats or bends to pick up things (G) | 24.9 (1.3) | 13/56 | 23 | 26 | 31/50 | 62 |  | 24.9 (1.2) | 179/7,810 | 2.3 | 2.5 |  | 13.8 (7.3-26.0)*** |
| 36 | Says ‘sentences’ of three or more words (C) | 36.7 (1.2) | 9/57 | 16 | 19 |  |  |  | 36.9 (1.0) | 483/7,615 | 6.3 | 6.7 |  | 3.3 (1.7-6.2)*** |
| 36 | Speech is understood by acquaintances (C) | 36.7 (1.1) | 9/50 | 18 | 22 |  |  |  | 37.0 (1.0) | 449/7,372 | 6.1 | 6.9 |  | 3.9 (2.0-7.4)*** |
| 36 | Rides (tri)cycle (G) | 36.6 (1.2) | 35/49 | 71 | 67 | 31/60 | 52 |  | 37.0 (1.0) | 990/5,893 | 16.8 | 17.6 |  | 9.6 (5.4-16.9)*** |
| 36 | Imitates drawing a vertical line (F) | 36.7 (1.2) | 16/48 | 33 | 37 | 9/41 | 22 |  | 37.0 (1.0) | 596/6,409 | 9.3 | 9.8 |  | 5.4 (3.1-9.5)*** |
| 36 | Walks smoothly (G) | 36.4 (1.3) | 17/57 | 30 | 34 |  |  |  | 36.9 (1.0) | 6/7,217 | 0.1 | 0.1 |  | 376 (127-1109)*** |

^a^Age in months (mo) at scheduled visits. ^b^Developmental domains: C, communication; F, fine motor activity, adaptive behaviour, and personal/social behaviour; G, gross motor activity. ^c^For each milestone, the category ‘I don’t know’ was removed from analysis. ^d^Results are based on logistic regression analyses with milestone attainment (yes/no) as the dependent variable and DMD (yes/no) as the independent variable. ^**^p<0.05.  ^**^p<0.01. ^***^p<0.001. OR, odds ratio; CI, confidence interval. Columns 1-5, 9-11 are adapted from ‘van Dommelen P, van Dijk O,  Wilde JA, Verkerk PH. Early developmental milestones in Duchenne muscular dystrophy. Dev Med Child Neurol 2020;62: 1198-1204’.
